# Brain-computer interfaces in pediatric populations: research advances, application challenges, and future directions

**DOI:** 10.3389/fped.2026.1936801

**Published:** 2026-09-29

**Authors:** Shan Wang, Qian Wu, Qiaorong Lei, Chunyang Li, Fenfang Lei

**Affiliations:** 1 School of Nursing, Shaoyang University, Shaoyang, Hunan, China; 2 Department of Science, Research and Education, Jiujiang Maternal and Child Health Hospital, Jiujiang, Jiangxi, China; 3 Department of Child Health Care, The First Affiliated Hospital of Shaoyang University, Shaoyang, Hunan, China

**Keywords:** brain-computer interface, children, clinical translation, developmental disorders, neurorehabilitation

## Abstract

Brain-computer interfaces (BCIs) establish direct communication pathways between the brain and external devices. Leveraging the unique neuroplasticity of the developing brain, BCIs have emerged as a novel rehabilitative technology for children with neurodevelopmental disorders, including cerebral palsy, autism spectrum disorder (ASD), attention deficit hyperactivity disorder (ADHD), and perinatal brain injury. This review comprehensively examines five major BCI paradigms—P300, motor imagery (MI), steady-state visual evoked potentials (SSVEP), functional near-infrared spectroscopy (fNIRS), and multimodal hybrid systems—with respect to their pediatric adaptability, technical limitations, and optimization strategies. We summarize the existing evidence across four key clinical applications: augmentative and alternative communication (AAC), motor function rehabilitation, ADHD attention training, and ASD social–emotional intervention. Key intrinsic (cognitive and physiological) and extrinsic (device and environmental) variables affecting BCI performance in children are analyzed, along with general optimization approaches including personalized parameter tuning, pre-training neurofeedback, and lightweight hardware modifications. Furthermore, we examine multilayer barriers to pediatric BCI adoption, including user experience, clinical workforce readiness, translational pathways, and child-specific ethical considerations. Preliminary evidence suggests that non-invasive BCIs can be feasibly used in children above 5 years of age, although this finding is based on short-term studies with small sample sizes and requires further validation through larger longitudinal trials. However, the immature pediatric brain, short attention spans, and low device tolerance preclude direct translation of adult BCI systems. While evidence is more established for AAC and motor rehabilitation in cerebral palsy, large-scale long-term data are still lacking for ADHD and ASD interventions. Future efforts should prioritize child-specific lightweight wearable devices, multicenter longitudinal trials, immersive integration with virtual reality (VR) and social robots, standardized clinical service frameworks, and ethical guidelines for invasive pediatric BCIs, thereby facilitating the transition of BCI technology from laboratory to clinical and home settings and maximizing its value in promoting neurodevelopmental plasticity in children with special needs.

## 1 Introduction

Brain-computer interfaces (BCIs) bypass peripheral nerves and muscles to establish bidirectional information pathways directly between the brain and external devices. A typical BCI system comprises four modules: signal acquisition, neural feature decoding, command output, and real-time feedback, forming a closed-loop control system. In pediatric populations, non-invasive electroencephalography (EEG)-based BCIs have become the predominant research modality for clinical rehabilitation and neurodevelopmental intervention, owing to their non-invasiveness, portability, and cost-effectiveness.

The pediatric brain undergoes continuous dynamic development from embryogenesis through adulthood, encompassing neuronal proliferation, migration, synaptic pruning, and white matter myelination. Newborns already possess nearly 100 billion neurons, but fine construction of neural circuits has not yet commenced. Synaptic density peaks between 3 and 6 years of age in most cortical regions, with some regional variation extending to 8 years, while prefrontal cortical maturation extends until the end of adolescence, and myelination continues throughout the school-age year (1). This unique developmental trajectory directly contributes to lower EEG signal-to-noise ratios, poorer rhythm stability, substantial inter-individual variability, and age-, sex-, and developmental rate-dependent signal changes in children. Nonetheless, the accompanying neuroplasticity provides a critical intervention window for children with neurological impairments and developmental disorders. For children with cerebral palsy, perinatal brain injury, ASD, ADHD, and other special conditions, BCIs can directly decode internal neural intentions into executable commands (2), thereby guiding targeted brain functional reorganization during the optimal plasticity period—an intervention approach that traditional rehabilitation modalities cannot readily replace. Existing clinical feasibility studies have demonstrated that children above 5 years of age can adapt to mainstream BCI paradigms including P300, motor imagery (MI), and steady-state visual evoked potentials (SSVEP) in short-term protocols (3–6). Short-term safety is favorable, and family acceptance continues to increase. Clinical applications now span AAC, motor rehabilitation for cerebral palsy, ADHD attention training, ASD social–emotional intervention, and communication support for children with locked-in syndrome. Research paradigms have evolved from early single-case explorations to small-sample trials, predominantly conducted at single centers, covering functional compensation, cognitive training, environmental control, and entertainment interactions. However, pediatric-specific characteristics—including limited attention, immature cognitive capacity, and low device tolerance (7, 8)—pose persistent challenges including insufficient technical adaptability, limited clinical evidence, absence of translational infrastructure, and underdeveloped ethical frameworks.

While several prior reviews have addressed adult BCI applications, and one pediatric-focused review by Orlandi et al. (2) examined BCIs for children with complex communication needs and limited mobility, no review has comprehensively examined all five major non-invasive BCI paradigms across the full range of pediatric clinical domains while systematically analyzing the pediatric-specific variables, optimization strategies, and translational barriers. Several recent systematic and scoping reviews have further advanced the field. Alexopoulou et al. (9) synthesized BCI-VR protocols for school-aged children with ASD; Raza et al. (10) provided a meta-analysis of ADHD attention-training; and Zhang et al. (11) conducted a comprehensive review of noninvasive BCI for children with ADHD and ASD across 24 RCTs. Our review differs from and adds to these contributions in three ways: (1) it covers all five major BCI paradigms (P300, MI, SSVEP, fNIRS, and hybrid) within a single framework, whereas prior reviews typically focused on single paradigms or single clinical conditions; (2) it explicitly analyzes pediatric-specific variables (intrinsic cognitive/physiological and extrinsic device/environmental factors) and their optimization strategies, which are often treated separately in other reviews; and (3) it integrates technical adaptation, clinical evidence, user experience, and full-chain translational barriers into a unified framework, providing a more comprehensive roadmap for clinical implementation. To clearly illustrate how this review differs from and adds to prior work, Table 1 summarizes these comparisons and the specific gaps addressed by the present review.

**Table 1:** Comparison of the present review with recent pediatric BCI reviews.

Review	Focus Area	Populations Covered	Paradigms Covered	Pediatric-Specific Variable & Optimization Analysis	Translational Barriers & Ethics	Gaps	Contribution of This Review
Orlandi et al. (2) (2021), systematic review	BCI for children with complex communication needs and limited mobility	Children with communication/mobility impairments	Mainly P300/access paradigms	Not systematically analyzed	Not addressed	Restricted to communication/mobility domain; single-paradigm emphasis	Expands to all four clinical domains and all five paradigms
Alexopoulou et al. (9) (2025), systematic review	BCI-VR protocols for school-aged children with ASD	ASD only	BCI instances within VR protocols	Partial (VR-related variables only)	Not addressed	Single population (ASD) and single integration modality (VR)	Covers ASD/ADHD plus AAC/motor domains in one framework
Raza et al. (10) (2025), meta-analysis	BCI-based attention-training for ADHD	ADHD only	Attention-training paradigms	Not systematically analyzed	Not addressed	Single clinical domain (ADHD attention) and meta-analytic synthesis only	Adds multi-domain evidence and full-chain analysis
Zhang et al. (11) (2025), comprehensive review (24 RCTs)	Noninvasive BCI for children with ADHD and ASD	ADHD and ASD	Noninvasive EEG paradigms	Not systematically analyzed across modalities	Limited (not full-chain)	Two disorders only; variable/optimization and translational/ethical dimensions treated separately	Unifies all five paradigms, pediatric variables & optimization, and translational/ethical barriers
This review	All five major non-invasive BCI paradigms across the full pediatric clinical continuum	CP, perinatal brain injury, ASD, ADHD, and other neurodevelopmental disorders	P300, MI, SSVEP, fNIRS, and hybrid	Comprehensively analyzed (intrinsic cognitive/physiological and extrinsic device/environmental factors+optimization strategies)	Comprehensively addressed (user experience, workforce readiness, knowledge translation, child-specific ethics)	—	Fills the three gaps above: five-paradigm framework, pediatric-specific variable/optimization analysis, and integrated translational–ethical roadmap

In the “Gaps” column, the dash (—) for “This review” indicates that this review is positioned to fill the gaps identified for the prior reviews, rather than to identify new gaps.

To fill this gap, the present review aims to: (1) examine the adaptability and limitations of five major BCI paradigms in pediatric populations; (2) summarize the clinical evidence across AAC, motor rehabilitation, ADHD attention training, and ASD social-emotional intervention; (3) analyze intrinsic and extrinsic variables affecting pediatric BCI performance and identify optimization strategies; and (4) discuss user experience, ethical considerations, and full-chain barriers to clinical translation. This narrative review aims to provide a theoretical foundation and practical reference for the development of pediatric neurorehabilitation devices and clinical intervention protocols.

## 2 Common BCI paradigms in children

This review is a narrative review; it was not conducted as a formal systematic or scoping review and therefore does not follow PRISMA or PRISMA-ScR guidelines. We searched PubMed, Web of Science, and IEEE Xplore databases for peer-reviewed original research, reviews, and clinical trials published up to August 2026. The specific search strategies employed for each database were as follows. For PubMed: (“brain-computer interface” OR “BCI” OR “brain-machine interface”) AND (“child*” OR “pediatric” OR “adolescent”) AND (“P300” OR “motor imagery” OR “SSVEP” OR “fNIRS” OR “hybrid BCI”) AND (“cerebral palsy” OR “autism” OR “ASD” OR “ADHD” OR “attention deficit” OR “neurodevelopmental disorders”), with filters for humans and English language. For Web of Science: same Boolean combinations with topic search (TS) and refined by document types (articles and reviews). For IEEE Xplore: (“brain-computer interface” OR “BCI”) AND (“child*” OR “pediatric”) with metadata only and full-text availability. The final search was executed on August 10, 2026, and results were exported to EndNote for screening.

Given the narrative nature of this review, study selection and data extraction were performed by a single reviewer (the lead author) with cross-verification by the corresponding author; disagreements, if any, were resolved through discussion. Extracted variables included study design, sample size, participant age range, BCI paradigm, outcome measures, and key quantified results. We did not perform formal quality scoring, but study designs and sample sizes are explicitly reported in Table 2 to allow readers to assess the evidence level of each cited study. Adult-only studies were excluded unless cited for methodological insight, in which case clear caveats were added. Conference abstracts and non-peer-reviewed reports were excluded, while full-length peer-reviewed conference papers (e.g., IEEE EMBC proceedings) were retained when they provided pediatric evidence not otherwise available in journal form. References were selected for their representativeness and relevance to the defined clinical domains.

**Table 2:** Study-level evidence summary for major pediatric BCI paradigms.

Paradigm	Representative Studies	Population	Sample Size (Pediatric)	Age Range	Key Quantified Outcomes	Evidence Quality
P300-BCI	Keough et al. (8); Pitt et al. (12); Jadavji et al. (3); Peketi & Dhok (14)	Pediatric	12–45 per study	5–17 years^a^	Kappa >0.4 (perinatal strok e^3^); 91.12% offline accuracy (ASD 14)	Pilot studies; case series
MI-BCI	Jadavji et al. (4); Behboodi et al. (15); Emami & Chau (17)	Pediatric	9–18 per study	6–18 years	73–78% classification accuracy (4, 15)	Pilot RCTs; feasibility studies
SSVEP-BCI	Schrag et al. (19); Brickwedde et al. (20) (adult proof-of-concept); Norton et al. (18) (pediatric time-window)	Mixed (pediatric+1 adult proof of concept)	8–35 per study	5–18 years^a^	>60% game control accuracy (high-frequency 20); 1–2 s optimal window (18)	Proof-of-concept; adult data extrapolation
fNIRS-BCI	Floreani et al. (22); Yang et al. (23)	Pediatric	10–30 per study	7–14 years	Age-correlated recognition accuracy improvement (22)	Exploratory studies
Hybrid BCI	Orlandi et al. (2) (review); multiple single-study reports	Mixed-age (pediatric-focused review)	Variable	5–18 years	Multi-scenario compatibility	Early-stage; limited pediatric data

a All cited studies are pediatric unless explicitly noted as “adult proof-of-concept” (Brickwedde et al. 20). Norton et al. (18), which provides the 1–2 s time-window finding, was conducted in 9–11-year-old children.

In this review, a BCI is defined as a system that acquires brain signals, decodes the user's intent, and translates that intent into an external command or feedback in real time, thereby forming a closed loop without relying on peripheral nerves and muscles. BCI paradigms (P300, motor imagery, and SSVEP) refer to the specific neural responses or mental strategies used to generate control signals, whereas signal-acquisition modalities refer to the physiological technique used to record brain activity, such as EEG or fNIRS. fNIRS is therefore treated here as a signal-acquisition modality rather than a separate control paradigm, and hybrid BCIs combine multiple paradigms or modalities. Neurofeedback (e.g., in ADHD training) is considered a BCI-based application in which the decoded signal is used to drive self-regulation rather than an external device, whereas diagnostic applications (e.g., emotion or attention monitoring) use the decoded signal for assessment without a control loop. The functional performance of a BCI system depends on specific neural signal paradigms. In pediatric populations, due to immature brain development, shorter attention spans, and individual variability in tolerance to visual stimuli, different paradigms exhibit performance and adaptability characteristics distinct from those observed in adults. This section sequentially reviews the application evidence, optimization explorations, and limitations of P300, MI, SSVEP, fNIRS, and multimodal hybrid BCIs in children.

### 2.1 P300 event-related potential BCI

P300-based BCIs (P300-BCIs) utilize the oddball paradigm to elicit a positive event-related potential at approximately 300 ms. By detecting this potential, the system determines which option the child is attending to, thereby enabling character selection or command output. This paradigm requires minimal training and offers intuitive symbol selection, making it the most widely used BCI approach for AAC in children with severe motor and speech impairments. The visual stimulus presentation mode significantly affects P300 signal quality in children. Dynamic animations with behavioral feedback can substantially amplify P200/P300 amplitudes (8), rendering them more suitable for younger children compared to static scaling displays. Conversely, simultaneous highlighting of multiple symbols tends to distract children, reducing ERP signal-to-noise ratios and classification accuracy (12).

Cognitive fatigue and working memory constraints represent the most prominent challenges for this paradigm in children. Sustained P300 tasks exceeding 30 min induce marked cognitive fatigue, manifested by elevated alpha-band power and attenuated attention efficiency (8). Children with perinatal stroke can achieve discrimination coefficients (kappa > 0.4) comparable to healthy peers in simple P300 tasks (3), suggesting intact basic signaling pathways and a foundation for communication intervention. At the same time, uneven working memory development in children—particularly in younger or cognitively delayed individuals—renders complex character input tasks difficult, with substantial inter-individual performance differences (13), a relationship established in adults that is expected to be amplified in the developing brain. Pre-screening for working memory is recommended before clinical adaptation, and for children with limited capacity, supportive strategies such as segmented input and visual memory cues should be employed. For children with ASD, who exhibit reduced amplitudes and prolonged latencies, adaptive variational mode decomposition algorithms can effectively optimize signal features, achieving offline classification accuracy of 91.12% (14), outperforming traditional linear discriminant analysis and temporal convolutional network models. However, this algorithm has only been validated offline, and its generalizability in real home or clinical settings requires large-scale verification.

Overall, P300-BCI enables stable character spelling in school-aged children, with core challenges being high cognitive load and visual fatigue. Future optimizations should focus on animated stimuli, short segmented tasks, and pre-implementation cognitive assessments, along with dedicated adaptive signal processing algorithms for ASD children to improve recognition performance.

### 2.2 Motor imagery (MI)-based BCI

Motor imagery BCIs (MI-BCIs) do not require external visual stimuli; instead, they rely on modulation of sensorimotor rhythms (SMR) to generate control signals, avoiding visual load and thus being more suitable for motor rehabilitation in children with cerebral palsy. MI-BCI is often combined with functional electrical stimulation (FES): by imagining grasping or wrist extension, the system triggers peripheral electrical stimulation to reconstruct motor feedback pathways. Existing clinical trials show that average classification accuracy in children with cerebral palsy using BCI-FES ranges between 73% and 78% in two pilot studies (4, 15), suggesting preliminary feasibility rather than established stability, with generally good tolerability. The major drawback of this paradigm lies in children's limited capacity for abstract imagination: they often struggle to understand the task logic of “simulating movement in the mind without actually moving the limbs,” resulting in substantially longer training periods than in adults and wide performance variability. A stereoelectroencephalography controlled trial which is cited here for its behavioral insight rather than as representative of the non-invasive approach indicated that only a minority of children can independently perform effective motor imagery control, whereas substituting actual limb movement guidance significantly improves recognition success (16). Moreover, environmental visual distractors increase cognitive load (elevated theta/alpha ratio), with children who have poorer baseline performance being more susceptible to interference (17), an effect first characterized in adults but expected to be more pronounced in pediatric users, thus necessitating maximal simplification of irrelevant visual elements in clinical training environments.

In summary, MI-BCI is dedicated to motor rehabilitation with no visual stimulation burden; optimization priorities include simplified training environments, pre-training with physical movement guidance to lower cognitive barriers, and personalized parameter tuning tailored to pediatric physiological characteristics. For MI-BCI specifically, time-window optimization should balance real-time responsiveness with classification stability, and adaptive window adjustment based on individual children's performance may be particularly beneficial for this paradigm—an area requiring further pediatric-specific investigation.

### 2.3 Steady-state visual evoked potential (SSVEP)-based BCI

SSVEP-based BCIs use periodic visual stimuli to elicit synchronized EEG rhythms, and with their minimal training requirements and relatively high information transfer rates, they hold certain application potential in pediatric populations. A pediatric SSVEP study in 9–11-year-old children further demonstrated that a short time window on the order of 1–2 s (with a minimum classification window of 1.25 s and a typical target-selection latency of ∼2 s) is sufficient to accommodate children's brief attention spans while preserving both real-time responsiveness and classification accuracy (18). However, compared to high-frequency stimulation paradigms, conventional low-frequency flickering stimuli tend to induce visual fatigue, dizziness, and poor training compliance in children, precluding long-term rehabilitation intervention. Two mainstream optimization pathways currently exist: first, texture-based replacement of flickering stimuli, which significantly improves subjective comfort in children within the 9–33 Hz range but does not notably enhance EEG signal-to-noise ratio or classification accuracy (19), indicating a trade-off between comfort and system performance; second, a proof-of-concept adult MEG study (20)demonstrated that invisible high-frequency tagging (56–60 Hz) could enable stable game control, suggesting a promising direction that warrants future pediatric validation. However, a notable research gap exists: long-term safety data regarding high-frequency stimulation on the developing retina and visual cortex remain unavailable, limiting large-scale clinical deployment. Therefore, while SSVEP-BCI offers outstanding interaction speed in children, its core limitation is low comfort with conventional stimulation. The current optimal solution is high-frequency imperceptible stimulation, and future work must prioritize long-term developmental safety verification.

### 2.4 fNIRS-based BCI and emotion recognition paradigm

fNIRS-based BCIs have recently gained attention in emotion recognition. By measuring changes in oxygenated and deoxygenated hemoglobin concentrations in the cerebral cortex, fNIRS reflects neural activity. Its main advantages over EEG are robustness to head movement artifacts and low wearing burden, making it suitable for children with hyperactivity or tactile sensitivity. Applications primarily focus on attention monitoring, emotion recognition, and early screening of developmental disorders. Adult multimodal controlled studies have demonstrated that dynamic video stimuli elicit stronger cortical responses than static images, and fNIRS combined with EEG yields significantly higher emotion classification accuracy than either modality alone (21). A dedicated study in healthy children aged 8–14 years showed that fNIRS can precisely capture neural activity and emotional states via prefrontal hemoglobin changes, with recognition accuracy improving with age (22). Deep learning-based fNIRS models can provide passive communication channels for children with low functional abilities and enable early risk warning for developmental issues such as dyslexia and ADHD (23). The main limitation of this paradigm is its low temporal resolution, which precludes capture of millisecond-scale rapid neural potentials, and single-modality accuracy remains limited—thus it is often combined with EEG signals. fNIRS-BCI offers the best wearing adaptability and is suitable for passive monitoring and intervention in children with severe disabilities, but its clinical stability requires large-sample validation.

### 2.5 Hybrid BCI designs for children

Given that each single BCI paradigm has inherent limitations in pediatric applications—such as the high learning curve of MI-BCI and the visual fatigue induced by P300 and SSVEP—recent research has begun exploring multimodal hybrid BCI schemes. By fusing multiple neural signal features or incorporating external inputs, these approaches aim to maintain or enhance control performance while reducing system complexity.

Because children differ markedly from adults in cognitive load, attention duration, and task comprehension, pediatric hybrid BCI design must strictly adhere to the “minimal cognitive load” principle (2). Hybrid BCIs optimize child–machine interaction via signal fusion and state monitoring, with a core principle of time-division multimodal switching to avoid parallel operations that increase cognitive pressure. Two mainstream combination strategies exist. The first is active control fusion, using MI as the primary motor control channel and P300 as the fine-command confirmation channel, supplemented by eye-tracking for command correction, thereby improving control stability without increasing training difficulty. The second is passive monitoring assistance, integrating fNIRS with EEG attention metrics (e.g., theta/beta ratio, TBR) into the main system to recognize fatigue or distraction in real time and automatically adjust task difficulty—a promising approach for ADHD and learning-disabled children. Studies indicate that ADHD patients exhibit elevated theta power and TBR during Go/No-Go tasks, particularly over frontal, temporal, and occipital regions (24); machine learning classifiers based on TBR can effectively distinguish ADHD patients from healthy controls, suggesting TBR's feasibility as a real-time attention assessment index for BCI systems. However, the practical obstacles of hybrid architectures center on hardware cost and system complexity: multi-sensor synchronous acquisition devices are expensive, and data synchronization and feature fusion algorithm debugging are technically demanding, placing them beyond the reach of primary rehabilitation facilities and ordinary families. Lightweight, low-cost systems represent the primary optimization direction.

### 2.6 Summary of paradigm clinical applicability

The five major BCI paradigms each have distinct applicable scenarios and limitations in children (Tables 2 and 3).

**Table 3:** Qualitative comparison of advantages, challenges, and optimization strategies for pediatric BCI paradigms.

Paradigm	Advantages	Pediatric-Specific Challenges	Optimization Strategies
P300-BCI	Mature symbol selection, no training required	High working memory demand, fatigue with prolonged tasks	Dynamic animated stimuli (8), segmented spelling, pre-training cognitive assessment (13)
MI-BCI	No visual stimulation, suitable for motor rehabilitation	Difficulty with abstract imagination, large individual variability	Time-window optimization balancing responsiveness and stability (pediatric-specific investigation needed); reduce visual distractors (17)
SSVEP-BCI	High information transfer rate, minimal training	Flicker-induced visual fatigue	Texture-based stimuli (19), high-frequency invisible tagging (pediatric validation needed 20); 1–2 s time window (18)
fNIRS-BCI	Portable, motion-artifact resistant	Low temporal resolution, limited accuracy alone	Fusion with EEG (21), deep learning feature extraction (23)
Hybrid/Passive BCI	Multi-scenario compatibility, real-time fatigue/attention monitoring	High device complexity and hardware cost	Sequential operation mode, phased modality switching, simplified sensor combinations (2)

These technical characteristics determine their clinical emphasis across different pediatric populations: P300-BCI is suitable for AAC in children with severe speech impairment; MI-BCI is more appropriate for motor rehabilitation in cerebral palsy and perinatal stroke; SSVEP-BCI suits high-speed interactive training in children with good tolerance; fNIRS-BCI targets severely disabled children unable to perform active tasks; and hybrid BCIs are more adaptable for attention-deficit populations such as ADHD and ASD, though their clinical efficacy remains insufficiently supported by evidence.

## 3 Four major clinical application scenarios of pediatric BCI

Based on the technical features of different BCI paradigms and following a logical progression of “functional compensation → motor remodeling → cognitive correction → social–emotional intervention,” this section reviews the clinical application status of BCI in AAC, motor rehabilitation and independent mobility, ADHD attention training, and ASD social–emotional intervention.

### 3.1 Augmentative and alternative communication (AAC): a fundamental expression channel for severely disabled children

For children with severe speech and physical disabilities—such as those with severe cerebral palsy, locked-in syndrome, or perinatal brain injury—who cannot communicate through gestures, speech, or touchscreens, establishing effective communication channels is the most basic functional need. BCI-AAC is the only communication means independent of peripheral muscles and represents the most extensively studied pediatric BCI application.

Parental acceptance is relatively high, with some families even preferring implantable electrodes despite higher risks, while showing no clear preference between visual and motor imagery control modalities (25). This technology can also be extended to mildly impaired children with touch capabilities to identify functional intentions and emotional states (26), and emerging affective BCI approaches may potentially assist in detecting internal emotional states to compensate for expressive language deficits. The greatest bottleneck for clinical translation is not algorithmic performance but the absence of clinical service infrastructure. Speech-language pathologists (SLPs) are the core practitioners for AAC intervention, yet surveys indicate that most clinicians lack training in BCI system fitting and personalized protocol design (27), with no standardized training system available. Frontline therapists prefer hands-on demonstrations, case-based teaching, and multimedia practical courses. Therefore, future technology development must incorporate stratified healthcare training programs to bridge the gap from laboratory to clinic.

### 3.2 Motor rehabilitation and independent mobility: neuroplasticity-based remodeling intervention

For children with motor impairments such as cerebral palsy and perinatal stroke, BCI-driven FES (BCI-FES) and wheelchair control systems offer potential pathways for active participation in rehabilitation training and autonomous mobility. BCI-FES combined with MI training constitutes the core approach for motor function remodeling in cerebral palsy and hemiparesis, promoting repair of damaged motor networks through active brain engagement, which offers potential advantages over conventional passive electrical stimulation based on preliminary evidence. A 6-week randomized controlled trial demonstrated that BCI-coupled electrical stimulation simultaneously enhanced prefrontal sensorimotor rhythms and beta-band neural activity, an effect not observed with electrical stimulation alone (28), suggesting that active brain participation may be an important driver of neural remodeling. A game-based visual feedback MI-BCI rehabilitation system, after 12–60 days of continuous intervention, increased clustering coefficients and shortened information transmission pathways in motor networks of children with cerebral palsy, indicating improved motor regulation efficiency (29). Two studies from one research group have shown that children with tetraplegic cerebral palsy can use BCI to control powered wheelchairs, with basic commands such as forward movement and turning achievable, though maintaining stationary control remains more challenging (5, 6). After 12 standardized training sessions, children showed significant improvements in occupational performance and goal attainment, though some experienced frustration from control errors during long-term training, reducing adherence (30). These short-term trials suggest that standardized training can enhance limb regulation and optimize brain network transmission efficiency in children, while also supporting wheelchair control for tetraplegic children to rebuild basic autonomous mobility. However, most current studies are single-center small-sample trials with short follow-up, lacking differentiated intervention protocols by age and condition type; the durability of long-term rehabilitation outcomes still requires high-level clinical evidence. Additionally, frustration during training tends to reduce long-term adherence, necessitating optimized feedback mechanisms to improve engagement.

### 3.3 ADHD attention training: cognitive enhancement intervention

Unlike applications that replace lost functions, BCI for ADHD serves as a cognitive remodeling tool. Leveraging the characteristic EEG features of ADHD—enhanced theta power, reduced beta power, and elevated TBR, particularly over frontal regions (24) —neurofeedback training guides children to self-regulate EEG rhythms, ameliorating core symptoms of inattention and impulsivity, and helping children learn self-regulation of attention.

Existing controlled trials demonstrate that lightweight home-based BCI training has been reported to produce effects comparable to traditional behavioral interventions in preliminary studies (31), and combined with pharmacotherapy has been reported to yield optimal cognitive improvement (32); gamified adaptive training significantly enhances attention levels in children (33). A recent meta-analysis (10) further quantified the efficacy of BCI attention-training for ADHD, providing meta-analytic evidence that complements the individual trial findings. A case study of an adolescent with ASD and comorbid ADHD showed that recreational BCI training could improve social initiative (34), though single-case findings lack generalizability. Mechanistically, current evidence suffers from a population gap: frontoparietal network regulation studies have been conducted primarily in healthy young adults, showing that even a single short session can optimize cortical information connectivity (35), but whether this mechanism can be replicated in the developing child brain requires dedicated controlled trials. A comprehensive review (11) synthesized evidence from 24 RCTs involving approximately 2,000 children with ASD and ADHD, providing a broader quantitative context for the findings discussed here. BCI combined with VR immersive training can enhance engagement, focus, and attention in ADHD populations (10, 11, 36), though most existing participants are university students, and systematic controlled studies on visual load, scenario adaptability, and long-term intervention effects in younger children are still lacking.

More recent 2025–2026 evidence should also be interpreted with caution: a double-blind, sham-controlled randomized trial of theta–beta mobile neurofeedback in 139 children with ADHD or neurotypical development found that active neurofeedback produced measurable neurophysiological modulation (increased theta–gamma coupling in frontal regions) yet was not superior to sham feedback for alleviating ADHD symptoms on clinical and cognitive outcomes (37). This underscores that, although portable neurofeedback systems are feasible and increasingly used, their specific clinical benefit beyond placebo or non-specific effects has not been firmly established in blinded pediatric trials.

### 3.4 ASD and emotional intervention: BCI–VR integrated approaches

BCI applications in ASD primarily focus on neurofeedback training and social–emotional ability intervention, but with broader targets including attention orienting, emotion recognition, and joint attention—core social functions—aiming to promote social improvement from the neural activity level. Paradigms such as Mu-rhythm neurofeedback and P300 joint-attention training can ameliorate social avoidance and emotion recognition deficits, while virtual scenarios efficiently simulate social contexts to enhance training utility. Small-sample studies have confirmed that these approaches can improve social behavior and emotion regulation in children with ASD (38). BCI combined with immersive VR technology represents an innovative direction for ASD intervention; a review covering 9 studies and 77 ASD participants demonstrated that BCI–VR integrated interventions can simultaneously improve multidimensional deficits: virtual scenarios for eye contact and conversational logic training to reduce social avoidance; immersive emotional scenarios for emotion recognition and expression; P300 paradigms for joint attention training; and Mu rhythms for long-term neurofeedback (9). As noted above, Zhang et al. (11) provided broader meta-analytic evidence across 24 RCTs, further supporting the potential of these integrated approaches. An ongoing meta-analysis aims to integrate all randomized controlled trials and conduct stratified analyses by age, symptom severity, and intervention duration (39), which will provide higher-level evidence for clinical protocol development. Auditory BCI represents a frontier exploratory direction for objectively assessing higher-order auditory cortex recovery in children with cochlear implants (40), though it has not yet reached clinical trial stage. Overall, the evidence level in this domain remains low, with common issues including lack of blank controls and small sample sizes, and a shortage of large-sample meta-analyses and long-term follow-up data, precluding large-scale clinical deployment at present. Moreover, motion artifacts from tactile-sensitive children and other frontier applications such as auditory assessment remain at the laboratory exploration stage.

Since the search update, no large-sample 2025–2026 primary studies specifically advancing BCI-FES for cerebral palsy or P300-based AAC in children were identified, and these directions remain supported primarily by earlier pilot and feasibility work rather than recent randomized evidence. In summary, pediatric BCI applications have expanded from AAC to motor rehabilitation, ADHD attention training, and ASD social intervention, forming a clinical continuum from functional substitution to neural remodeling. There is relatively strong evidence supporting AAC and motor rehabilitation for cerebral palsy, permitting small-scale clinical implementation; home-based cognitive training for ADHD is developing rapidly yet lacks long-term efficacy data; immersive interventions for ASD show considerable innovation but are in urgent need of high-quality clinical evidence. All scenarios depend on personalized adaptation to optimize intervention outcomes.

## 4 Variables affecting pediatric BCI performance and optimization strategies

Inter-individual performance variability in children using BCI is substantially greater than in adults. Variables can be categorized into intrinsic (child-specific cognitive and physiological) factors and extrinsic (device and environmental) factors, with corresponding optimization approaches.

### 4.1 Intrinsic variables: individual child-related and state-dependent factors

Intrinsic variables affecting pediatric BCI performance include attention, working memory, motor imagery ability, motivation, and neurophysiological developmental characteristics. Attention is one of the core intrinsic variables; studies indicate that inattention is a primary reason for poor BCI performance in children, especially for P300 and SSVEP paradigms that depend on sustained visual attention (2, 41). The effective attention window in children is only 15–20 min; beyond this threshold, alpha activity increases and cognitive resources are progressively depleted, such that although children may appear to continue operating, internal recognition efficiency declines substantially (8). Clinical training should adopt a “short-session, high-frequency” schedule with rest and mildly engaging interactive breaks every 15 min. Working memory capacity directly determines performance in complex P300 spelling tasks, with higher-capacity users performing significantly better in complex tasks (13), a pattern demonstrated in adults and likely to apply with even greater variability in children. Children's memory capacity continues to develop with age and shows marked individual differentiation. Low-memory children require supportive measures such as segmented tasks and visual prompts, with brief memory screening recommended before clinical use. MI-BCI is highly dependent on abstract thinking (17); younger children should transition from actual movement observation and physical practice to pure imagination training. Frustration during long-term training can substantially reduce participation willingness (30), necessitating adaptive difficulty and positive feedback mechanisms to sustain motivation. The number of synaptic connections in children peaks between 3 and 8 years of age (1), and white matter myelination continues throughout childhood (42), resulting in lower EEG signal-to-noise ratios and poorer rhythm stability. A uniform algorithm cannot accommodate all children, and the system must have adaptive signal processing capabilities. Compared to adults, children exhibit greater variability across these dimensions and are in a state of dynamic change, imposing higher demands on BCI system adaptability.

### 4.2 Extrinsic variables: modifiable device and environmental conditions

Extrinsic variables affecting pediatric BCI performance include visual distractors, stimulus parameters, time windows, feedback modes, and device comfort. Children are highly sensitive to irrelevant images, voices, and environmental clutter; visual distractors increase cognitive load, with low-performing participants being most affected (17), as shown in adult studies and anticipated to be more marked in children. Training environments should minimize irrelevant visual stimuli, favoring plain backgrounds, simplified interfaces, and reduced movement of non-essential personnel. For P300, dynamic animated materials are preferred, while simultaneous highlighting of multiple symbols should be avoided (8, 12); for SSVEP, high-frequency invisible stimuli are preferred to balance comfort and accuracy (19, 20). A moderate 1–2 s window is generally recommended for pediatric SSVEP-BCI (18), and similar considerations of short time windows may also apply to other paradigms such as MI-BCI to accommodate children's shorter attention spans. Gamified visual feedback and staged rewards significantly enhance pediatric adherence (4, 8, 29); long continuous sessions designed for adults should be replaced by multiple short training periods. Children have smaller head sizes and are more active; wet electrodes are cumbersome to apply and cause scalp pressure, leading to resistance. Hardware optimization should focus on dry electrodes and flexible fabric headbands integrated into everyday accessories such as headbands or cartoon caps to reduce wearing aversion (41). These factors are largely modifiable through system design, but the optimization directions often differ from those for adults, requiring a starting point rooted in children's cognitive and physiological characteristics.

### 4.3 Optimization strategies

Abandon uniform fixed adult parameters and dynamically adjust system configuration based on child age, cognitive level, and real-time state: segment task difficulty according to working memory screening, adding memory aids for cognitively weaker children (13); monitor alpha-band fatigue indices in real time, automatically pausing training and switching to light entertainment tasks when fatigue accumulates (8); adjust stimulus flicker speed and animation complexity based on real-time attention levels (24); and adaptively switch the time-window length for SSVEP-BCI according to single-trial classification accuracy (18). Dedicated neurofeedback training can optimize children's baseline EEG signals, thereby fundamentally improving BCI performance: targeted training of parietal alpha rhythms can enhance SSVEP signal-to-noise ratio and information transfer rate while optimizing occipitoparietal information pathways (43); deep learning-based adaptive calibration algorithms have been shown to reduce initial calibration trials in pilot studies for MI-BCI, lowering the usage threshold, with offline classification accuracy reaching 80.8% in children with cerebral palsy (15); employ plain, interference-free training environments paired with child-specific flexible lightweight wearable devices to replace cumbersome traditional wet-electrode setups, reducing wearing discomfort and signal artifacts for both home and clinical use.

In summary, compared to adults, pediatric populations exhibit stronger inter-individual variability and dynamic changes across these dimensions, imposing higher demands on BCI system adaptability. Current mainstream optimization strategies include personalized parameter tuning and neurofeedback enhancement, both of which have shown positive effects in preliminary studies, though their long-term efficacy and broad applicability require further validation. Understanding these variables and their optimization ultimately aims to improve pediatric BCI user experience and facilitate clinical translation. Nevertheless, the journey from laboratory to real-world routine application still faces multiple barriers in terms of technical accessibility, user acceptance, clinician awareness, and ethical frameworks.

## 5 User experience, ethical considerations, and full-chain barriers to clinical translation

Whether BCI can move beyond the laboratory to benefit children depends not only on algorithmic and hardware performance, but also on whether users and families are willing to adopt it, whether clinicians can prescribe it, and whether the healthcare system has the corresponding support capacity. These are critical non-technical issues.

### 5.1 Child and family user experience

The home is the primary setting for pediatric BCI home-based intervention. BCI offers children with severe disabilities a novel experience of autonomous control, breaking the cycle of prolonged passive care and alleviating learned helplessness; parents generally acknowledge its value in improving children's social participation (44). However, four main issues persist with long-term use (45): conventional devices are cumbersome to wear with poor comfort, provoking strong resistance in young and tactile-sensitive children; training tasks are monotonous and repetitive, leading to poor long-term adherence; high device costs without insurance coverage impose substantial financial burdens; and home use lacks real-time professional guidance from rehabilitation therapists, making parameter adjustment difficult. Corresponding optimization directions for family-oriented pediatric BCI systems include developing low-cost consumer-grade lightweight devices, integrating immersive interactive scenarios, and establishing remote rehabilitation guidance platforms.

### 5.2 Clinician awareness and preparedness

Rehabilitation physicians, pediatricians, and speech-language therapists are the core gatekeepers for BCI referral and prescription. However, a Canadian national survey (46) reported that over 40% of clinicians are almost unfamiliar with BCI technology, and only 4% have practical experience with child BCI use. Most practitioners in pediatrics, rehabilitation, and speech therapy lack BCI-specific expertise and hands-on experience. The industry currently lacks standardized diagnostic and treatment pathways, assessment scales, intervention protocols, and insurance reimbursement codes, further impeding routine clinical adoption. Solutions include integrating BCI training into residency curricula, establishing regional pediatric BCI rehabilitation centers, and developing unified operational guidelines and assessment standards.

### 5.3 Barriers and facilitators for knowledge translation from laboratory to clinic

Despite the demonstrated potential of BCI in multiple pediatric studies, the “knowledge translation” process from research to routine clinical application faces systemic obstacles spanning technical, user, clinical, and policy dimensions. Technically, devices suffer from poor adaptability, dependence on specialized environments, and operational complexity. User-level issues include wearing discomfort, privacy concerns, and anxiety over device obsolescence. Clinically, there is insufficient workforce capacity and absent service frameworks, preventing independent BCI-AAC fitting (27, 46). Policy-wise, there are no dedicated regulatory guidelines, incomplete ethical review frameworks, and no insurance coverage. Gefen et al. (47) proposed a phased knowledge translation strategy: developing low-cost home-use lightweight devices; gamified adaptive training to boost adherence; establishing regional rehabilitation centers for tiered training of physicians and therapists; conducting health economics evaluations to promote insurance coverage; and involving children, families, and clinicians in device iteration from the early stages to align with real-world needs.

### 5.4 Pediatric-specific ethical risks

Children lack full informed consent capacity, and their brains are still developing, making the ethical risks of BCI applications considerably higher than in adults. Core ethical principles include: establishing dynamic assessment and graded usage mechanisms for children with disabilities, determining intervention suitability based on neurodevelopmental level (48); strictly restricting invasive BCI applications to high-benefit, severely affected children, with age-adaptive dynamic informed consent mechanisms (49); and balancing technological intervention with social inclusion—while optimizing BCI intervention, simultaneously improving barrier-free environments in schools and communities to avoid the limitations of a single-technology solution.

### 5.5 Quality appraisal and limitations of the evidence base

It is important to explicitly acknowledge the quality and limitations of the evidence synthesized in this review. First, as a narrative review, no formal systematic search strategy, quality appraisal, or risk-of-bias assessment was conducted; thus, the selection and interpretation of included studies may be subject to author bias. Second, much of the available evidence in pediatric BCI research derives from single-center pilot studies, case series, and small-sample trials with limited follow-up, and few high-quality multicenter randomized controlled trials exist across any of the four clinical domains reviewed. Third, several conclusions rely on findings from adult populations (e.g., time-window optimization) or single research groups (e.g., wheelchair control studies), limiting the generalizability of the corresponding recommendations. Fourth, this review did not perform quantitative meta-analysis due to heterogeneity in study populations, paradigms, and outcome measures; reported effect sizes are thus narrative rather than pooled. Fifth, the rapidly evolving nature of BCI hardware and algorithms means that recently published studies may have been missed. Despite these limitations, we believe this review provides a comprehensive and timely framework for understanding the current state and future directions of pediatric BCI research.

## 6 Conclusions and future perspectives

### 6.1 Key research conclusions

This review examines the major BCI technological paradigms and clinical applications in pediatric populations. The core conclusions are as follows: First, preliminary evidence from short-term feasibility studies suggests that children above 5 years of age with special developmental disabilities can use various non-invasive BCI technologies, with favorable short-term safety and family acceptance, which may facilitate functional compensation and brain functional remodeling; however, larger longitudinal studies are needed to confirm long-term safety and efficacy. Second, the unique physiological and cognitive characteristics of children preclude direct translation of adult BCI technologies; limited attention, poor signal stability, and high cognitive demands constitute the core pediatric adaptation challenges. Third, personalized parameter tuning, pre-training neurofeedback, and lightweight hardware modifications may help address technical adaptability and reduce inter-individual performance variability. Fourth, technology adoption is constrained by multiple barriers including workforce shortages, device costs, and policy/ethical factors, requiring multidimensional collaborative efforts to improve the translational ecosystem.

### 6.2 Future research directions

Based on current research gaps and translational needs, six core directions are proposed for future pediatric BCI research, with emphasis on feasibility and innovation:

**First, develop pediatric-specific ethical and indication guidelines.** Clarify the scope of application for invasive and non-invasive BCIs in children, establish age-stratified dynamic informed consent and risk assessment mechanisms (49), fill the gap in long-term safety data for pediatric implantable devices, and form standardized ethical review frameworks.

**Second, develop lightweight child-specific wearable devices.** Focus on the three core requirements of comfort, portability, and low cost; develop flexible dry electrodes and integrated everyday wearable devices; simplify operation procedures to accommodate long-term use across home, school, and clinical settings, addressing the core pain points of poor adaptability and low adherence (45).

**Third, advance immersive technology integration research.** Promote deep integration of BCI with VR and intelligent robots; optimize gamified, scenario-based intervention models to address the monotony and adherence issues of traditional training; focus on validating the long-term efficacy and developmental appropriateness of immersive interventions for ASD and ADHD (9, 36, 50).

**Fourth, conduct high-quality multicenter clinical studies.** Move beyond current small-sample, short-follow-up studies; conduct large-sample, long-term randomized controlled trials with stratified analyses by age and condition type; establish standardized intervention protocols, intensity parameters, and efficacy evaluation systems to elevate the level of clinical evidence.

**Fifth, establish standardized clinical service frameworks.** Integrate pediatric BCI technology into residency training for rehabilitation, pediatric, and speech-language therapy; develop unified standards for device fitting, intervention procedures, and outcome assessment; establish regional specialized rehabilitation centers to bridge the laboratory-to-clinic translational gap.

**Sixth, build a cognitive pre-training optimization system.** Targeting the individual variability in working memory and abstract imagination abilities in children, develop phased cognitive pre-training programs paired with personalized cognitive load reduction strategies, thereby fundamentally narrowing the performance gap in pediatric BCI control and enhancing technological accessibility.

We acknowledge several methodological limitations of this review itself. First, our search was restricted to English-language publications, which may have introduced language bias. Second, the narrative design—while appropriate for this emerging and heterogeneous field—does not permit quantitative synthesis or formal hypothesis testing. Third, the rapid pace of publication in pediatric BCI means that some recent studies may not have been captured within our search timeframe (up to August 2026). Fourth, our reliance on the available published literature inevitably inherits the publication bias of the field, where positive results are more likely to be reported than negative or null findings. Readers should therefore interpret our conclusions as a synthesis of the current evidence landscape rather than definitive practice recommendations.

In conclusion, pediatric BCI has evolved from a single device-control tool into a core neurodevelopmental intervention technology. Leveraging the advantages of neuroplasticity, it holds irreplaceable value in the rehabilitation of children with special needs. Future efforts should integrate technological iteration, clinical validation, system improvement, and ethical governance to facilitate the transition of BCI from laboratory to clinical and home settings, helping children with developmental disabilities overcome barriers and participate equally in social life.

## References

[1] Sultana OF Bandaru M Islam MA Reddy PH. Unraveling the complexity of human brain: structure, function in healthy and disease states. Ageing Res Rev. (2024) 100:102414. 10.1016/j.arr.2024.102414 39002647 PMC11384519

[2] Orlandi S House SC Karlsson P Saab R Chau T. Brain-computer interfaces for children with complex communication needs and limited mobility: a systematic review. Front Hum Neurosci. (2021) 15:643294. 10.3389/fnhum.2021.643294 34335203 PMC8319030

[3] Jadavji Z Zhang J Paffrath B Zewdie E Kirton A. Can children with perinatal stroke use a simple brain computer interface? Stroke. (2021) 52(7):2363–70. 10.1161/strokeaha.120.030596 34039029

[4] Jadavji Z Kirton A Metzler MJ Zewdie E. BCI-activated electrical stimulation in children with perinatal stroke and hemiparesis: a pilot study. Front Hum Neurosci. (2023) 17:1006242. 10.3389/fnhum.2023.1006242 37007682 PMC10063823

[5] Floreani ED Rowley D Kelly D Kinney-Lang E Kirton A. On the feasibility of simple brain-computer interface systems for enabling children with severe physical disabilities to explore independent movement. Front Hum Neurosci. (2022) 16:1007199. 10.3389/fnhum.2022.1007199 36337857 PMC9633669

[6] Floreani ED Rowley D Khan N Kelly D Robu I Kirton A. “Unlocking independence: exploring movement with brain-computer interface for children with severe physical disabilities”. In: 2021 43rd Annual International Conference of the IEEE Engineering in Medicine & Biology Society (EMBC), Piscataway, NJ: IEEE (2021). p. 5864–7. 10.1109/embc46164.2021.9630578 34892453

[7] Pitt KM Cole ZJ Zosky J. Promoting simple and engaging brain–computer interface designs for children by evaluating contrasting motion techniques. J Speech Lang Hear Res. (2023) 66(10):3974–87. 10.1044/2023_jslhr-23-00292 37696046

[8] Keough JRG Irvine B Kelly D Wrightson J Comaduran Marquez D Kinney-Lang E. Fatigue in children using motor imagery and P300 brain-computer interfaces. J Neuroeng Rehabil. (2024) 21(1):61. 10.1186/s12984-024-01349-2 38658998 PMC11040843

[9] Alexopoulou A Pergantis P Koutsojannis C Triantafillou V Drigas A. Non-invasive BCI-VR applied protocols as intervention paradigms on school-aged subjects with ASD: a systematic review. Sensors. (2025) 25(5):1342. 10.3390/s25051342 40096108 PMC11902634

[10] Raza MZ Omais M Arshad HME Maqsood M Nadeem AA. Effectiveness of brain-computer interface (BCI)-based attention training game system for symptom reduction, behavioral enhancement, and brain function modulation in children with ADHD: a systematic review and single-arm meta-analysis. NeuroRegulation. (2025) 12(1):51. 10.15540/nr.12.1.51

[11] Zhang T Zhou X Li X Wang Y Fan Q Liang J. Noninvasive brain–computer interfaces for children with neurodevelopmental disorders: attention deficit hyperactivity disorder and autism spectrum disorder. Displays. (2025) 86:102886. 10.1016/j.displa.2024.102886

[12] Pitt KM Spoor A Zosky J. Considering preferences, speed and the animation of multiple symbols in developing P300 brain-computer interface for children. Disabil Rehabil Assist Technol. (2024) 20(1):171–83. 10.1080/17483107.2024.2359479 38808372

[13] Sprague SA McBee MT Sellers EW. The effects of working memory on brain–computer interface performance. Clin Neurophysiol. (2016) 127(2):1331–41. 10.1016/j.clinph.2015.10.038 26620822 PMC4747807

[14] Peketi S Dhok SB. Machine learning enabled P300 classifier for autism spectrum disorder using adaptive signal decomposition. Brain Sci. (2023) 13(2):315. 10.3390/brainsci13020315 36831857 PMC9954262

[15] Behboodi A Kline J Gravunder A Phillips C Parker SM Damiano DL. Development and evaluation of a BCI-neurofeedback system with real-time EEG detection and electrical stimulation assistance during motor attempt for neurorehabilitation of children with cerebral palsy. Front Hum Neurosci. (2024) 18:1346050. 10.3389/fnhum.2024.1346050 38633751 PMC11021665

[16] A Jensen M Schalk G Ince N Hermes D Worrell GA Brunner P. sEEG-based brain-computer interfacing in a large adult and pediatric cohort. J Neural Eng. (2025) 22(6):066041. 10.1088/1741-2552/ae2955 PMC13127260 41360010

[17] Emami Z Chau T. The effects of visual distractors on cognitive load in a motor imagery brain-computer interface. Behav Brain Res. (2020) 378:112240. 10.1016/j.bbr.2019.112240 31614183

[18] Norton JJS Mullins J Alitz BE Bretl T. The performance of 9–11-year-old children using an SSVEP-based BCI for target selection. J Neural Eng. (2018) 15(5):056012. 10.1088/1741-2552/aacfdd 29952751 PMC6309895

[19] Schrag E Comaduran Marquez D Kirton A Kinney-Lang E. An investigation into the comfort and neural response of textured visual stimuli in pediatric SSVEP-based BCI. Sci Rep. (2025) 15(1):26168. 10.1038/s41598-025-09540-8 40681665 PMC12274615

[20] Brickwedde M Bezsudnova Y Kowalczyk A Jensen O Zhigalov A. Application of rapid invisible frequency tagging for brain computer interfaces. J Neurosci Methods. (2022) 382:109726. 10.1016/j.jneumeth.2022.109726 36228894 PMC7615063

[21] Sun Y Ayaz H Akansu AN. Multimodal affective state assessment using fNIRS+EEG and spontaneous facial expression. Brain Sci. (2020) 10(2):85. 10.3390/brainsci10020085 32041316 PMC7071625

[22] Floreani ED Orlandi S Chau T. A pediatric near-infrared spectroscopy brain-computer interface based on the detection of emotional valence. Front Hum Neurosci. (2022) 16:938708. 10.3389/fnhum.2022.938708 36211121 PMC9540519

[23] Yang P Duan Y Wang L Gao Y Zhang Y Liang Z. An early detection framework for young Chinese learners at risk of Reading difficulty using fNIRS and deep learning. Sci Rep. (2026) 16(1):14104. 10.1038/s41598-026-44379-7 41851364 PMC13136355

[24] Torgersen EL Ragnarson I Molinas M. “Decoding attention through EEG: paving the way for BCI applications in attention-related disorders”. In: 2025 47th Annual International Conference of the IEEE Engineering in Medicine and Biology Society (EMBC), Piscataway, NJ: IEEE (2025). p. 1–7. 10.1109/embc58623.2025.11252840 41336877

[25] Branco MP Verberne MSW Van Balen BJ Bekius A Leinders S Ketelaar M. Stakeholder’s perspective on brain–computer interfaces for children and young adults with cerebral palsy. Disabil Rehabil Assist Technol. (2025) 20(8):3201–11. 10.1080/17483107.2025.2481426 40122080

[26] Pitt KM Mikuls A Ousley CL Boster JB Mahmoudi M McCarthy J. Considering whether brain–computer interfaces have prospective potential to support children who have the physical abilities for touch-based AAC access: a forum manuscript. Augmentative and Alternative Communication. (2025) 42(1):5–13. 10.1080/07434618.2025.2495897 40454682 PMC12354177

[27] Pitt KM McKelvey M Weissling K Thiessen A. Brain-computer interface for augmentative and alternative communication access: the initial training needs and learning preferences of speech-language pathologists. Int J Speech Lang Pathol. (2024) 27(1):14–22. 10.1080/17549507.2024.2363939 39028220

[28] Kim TW Lee BH. Clinical usefulness of brain-computer interface-controlled functional electrical stimulation for improving brain activity in children with spastic cerebral palsy: a pilot randomized controlled trial. J Phys Ther Sci. (2016) 28(9):2491–4. 10.1589/jpts.28.2491 27799677 PMC5080159

[29] Xie J Jiang L Li Y Chen B Li F Jiang Y. Rehabilitation of motor function in children with cerebral palsy based on motor imagery. Cogn Neurodyn. (2021) 15(6):939–48. 10.1007/s11571-021-09672-3 34790263 PMC8572258

[30] Hammond L Rowley D Tuck C Floreani ED Wieler A Kim VS-H. BCI move: exploring pediatric BCI-controlled power mobility. Front Hum Neurosci. (2025) 19:1456692. 10.3389/fnhum.2025.1456692 40270567 PMC12015940

[31] Lim CG Soh CP Lim SSY Fung DSS Guan C Lee T-S. Home-based brain–computer interface attention training program for attention deficit hyperactivity disorder: a feasibility trial. Child Adolesc Psychiatry Ment Health. (2023) 17(1):15. 10.1186/s13034-022-00539-x 36698168 PMC9878772

[32] Turan S Çıray RO. Comparative effects of BCI-based attention training, methylphenidate, and citicoline on attention and executive function in school-age children: a quasi-experimental study. Medicina (B Aires). (2026) 62(3):448. 10.3390/medicina62030448 PMC13027574 41901531

[33] Yang W Yuan J Ding L Chow SKK. A neurofeedback-guided EEG and BCI framework for personalized attention rehabilitation in ADHD. Neuroscience. (2026) 602:67–80. 10.1016/j.neuroscience.2026.03.010 41831590

[34] Van Damme S Mumford L Johnson A Chau T. Case report: novel use of clinical brain-computer interfaces in recreation programming for an autistic adolescent with co-occurring attention deficit hyperactivity disorder. Front Hum Neurosci. (2024) 18:1434792. 10.3389/fnhum.2024.1434792 39296916 PMC11408342

[35] Huang Y Ding Q Chen Z Chen J Li Y Chen L. Brain–computer interface training enhances attention function via modulating frontoparietal connectivity: evidence from functional near-infrared spectroscopy. Neural Plast. (2026) 2026(1):8133428. 10.1155/np/8133428 41497491 PMC12767425

[36] Wolfers J Hurst W Krampe C. Integrating EEG sensors with virtual reality to support students with ADHD. Sensors. (2026) 26(3):1017. 10.3390/s26031017 41682533 PMC12900143

[37] Kim JW Kim B-N Kim J Yang C-M Kwon J. Effects of mobile neurofeedback on theta–gamma coupling and clinical outcomes in children with ADHD: a double-blind, sham-controlled randomized clinical trial. Neuropsychiatr Dis Treat. (2025) 21:2733–46. 10.2147/ndt.s554756 41368620 PMC12683163

[38] LaMarca K Gevirtz R Lincoln AJ Pineda JA. Brain–computer interface training of mu EEG rhythms in intellectually impaired children with autism: a feasibility case series. Appl Psychophysiol Biofeedback. (2023) 48(2):229–45. 10.1007/s10484-022-09576-w 36607454

[39] Zhu H Gan Y Ye J Li Y Yu JZ Li X. Effectiveness of brain-computer interface interventions in autism spectrum disorder rehabilitation: a systematic review and meta-analysis protocol. BMJ Open. (2026) 16(1):e102277. 10.1136/bmjopen-2025-102277 41529886 PMC12815232

[40] Zheng Q Wu Y Zhu J Feng K Bai Y Li G. Applications and challenges of auditory brain-computer interfaces in objective auditory assessments for pediatric cochlear implants. Exploration. (2025) 5(3):20240078. 10.1002/exp.20240078 40585760 PMC12199368

[41] Jadavji Z Zewdie E Kelly D Kinney-Lang E Robu I Kirton A. Establishing a clinical brain-computer interface program for children with severe neurological disabilities. Cureus. (2022) 14(6):e26215. 10.7759/cureus.26215 35891842 PMC9307353

[42] Camacho MC Quiñones-Camacho LE Perlman SB. Does the child brain rest?: an examination and interpretation of resting cognition in developmental cognitive neuroscience. NeuroImage. (2020) 212:116688. 10.1016/j.neuroimage.2020.116688 32114148 PMC7190083

[43] Sun J He J Gao X. Neurofeedback training of the control network in children improves brain computer interface performance. Neuroscience. (2021) 478:24–38. 10.1016/j.neuroscience.2021.08.010 34425160

[44] Van Damme S Mumford L Thompson A Murphy C Ali T Hegazi A. Family experiences in a pediatric clinical brain–computer interface program: a qualitative study. Am J Occup Ther. (2026) 80(3):531–41. 10.5014/ajot.2026.051474 41920177

[45] Siu C Aoude M Andersen J Adams KD. The lived experiences of play and the perspectives of disabled children and their parents surrounding brain-computer interfaces. Disabil Rehabil Assist Technol. (2024) 19(7):2641–50. 10.1080/17483107.2024.2333884 38533741

[46] Letourneau S Zewdie ET Jadavji Z Andersen J Burkholder LM Kirton A. Clinician awareness of brain computer interfaces: a Canadian national survey. J Neuroeng Rehabil. (2020) 17(1):2. 10.1186/s12984-019-0624-7 31907010 PMC6945584

[47] Gefen N Mazer B Krasovsky T Weiss PL. Novel rehabilitation technologies in pediatric rehabilitation: knowledge towards translation. Disabil Rehabil Assist Technol. (2024) 20(5):1209–18. 10.1080/17483107.2024.2445017 39727293

[48] Schiff ND Diringer M Diserens K Edlow BL Gosseries O Hill NJ. Brain–computer interfaces for communication in patients with disorders of consciousness: a gap analysis and scientific roadmap. Neurocrit Care. (2024) 41(1):129–45. 10.1007/s12028-023-01924-w 38286946 PMC11284251

[49] Bergeron D Iorio-Morin C Bonizzato M Lajoie G Orr Gaucher N Racine É. Use of invasive brain-computer interfaces in pediatric neurosurgery: technical and ethical considerations. J Child Neurol. (2023) 38(3-4):223–38. 10.1177/08830738231167736 37116888 PMC10226009

[50] Cervantes J-A López S Cervantes S Hernández A Duarte H. Social robots and brain–computer interface video games for dealing with attention deficit hyperactivity disorder: a systematic review. Brain Sci. (2023) 13(8):1172. 10.3390/brainsci13081172 37626528 PMC10452217

